# Supplementary material for: Characteristics of methane emissions from alpine thermokarst lakes on the Tibetan Plateau
Source: Nat Commun. 2023 May 30;14:3121. doi: 10.1038/s41467-023-38907-6 (PMC10229571; doi:10.1038/s41467-023-38907-6)
Supplement: Supplementary file 1 — Supplementary Information [file 41467_2023_38907_MOESM1_ESM.pdf]

Supplementary Information for

Characteristics of methane emissions from alpine thermokarst lakes on the Tibetan Plateau

Guibiao Yang[1], Zhihu Zheng[1,2], Benjamin W. Abbott[3], David Olefeldt[4], Christian Knoblauch[5], Yutong Song[1,2], Luyao Kang[1,2], Shuqi Qin[1], Yunfeng Peng[1] and Yuanhe Yang[1,2*]

[1]State Key Laboratory of Vegetation and Environmental Change, Institute of Botany, Chinese Academy of Sciences, Beijing 100093, China

[2]University of Chinese Academy of Sciences, Beijing 100049, China

[3]Department of Plant and Wildlife Sciences, Brigham Young University, Provo, Utah, USA

[4]Department of Renewable Resources, University of Alberta, Edmonton, Alberta T6G 2H1, Canada

[5]Institute of Soil Science, University of Hamburg, 20146 Hamburg, Germany

[*]**Corresponding author:** Dr. Yuanhe Yang, tel.: + 86 10-6283 6638, E-mail: yhyang@ibcas.ac.cn

Supplementary Note 1: CO_2 concentrations and fluxes in alpine thermokarst lakes

Both CO_2 concentrations and fluxes were determined simultaneously with CH_4 measurements. These measurements revealed that dissolved CO_2 concentrations did not have obvious seasonal differences, but varied between 16.0 and 34.7 μmol L^{-1} across the 30 sampling clusters with a mean value of 19.3 ± 0.7 μmol L^{-1} (n = 30; Supplementary Fig. 3b). 62% of the studied thermokarst lakes were supersaturated in CO_2 with respect to the local atmosphere (ranging from 89.9 to 206.4% with an average of 108.5 ± 3.9%; n = 30). CO_2 fluxes showed high spatial variability across the 30 clusters, ranging from -33.7 to 445.0 mmol m^{-2} d^{-1} with an average value of 170.4 ± 21.8 mmol m^{-2} d^{-1} (n = 30; Supplementary Fig. 9). Thermokarst lakes distributed in alpine meadow and swamp meadow had higher CO_2 fluxes than those located in alpine steppe (Supplementary Fig. 4c). However, no significant difference was detected between thermokarst lakes located in alpine meadow and swamp meadow. As a whole, we observed high CO_2 flux in alpine thermokarst lakes, with values being ~5-times greater than those (35.2 mmol m^{-2} d^{-1}) from small ponds (size class < 0.001 km^2)[1]. To obtain the regional estimation, we upscaled CO_2 fluxes from the lake level to the regional scale using the same approach as CH_4. The upscaling analyses demonstrated that CO_2 emissions from alpine thermokarst lakes on the Tibetan Plateau were 2084.7 Gg (10^9 g) CO_2 yr^{-1} (Table 1). Overall, despite emergent plants and their associated CO_2 uptake, alpine thermokarst lakes are still expected to a significant carbon source due to the sparsity of plants in most lakes (Supplementary Table 1).

Supplementary Note 2: Radiocarbon age measurements for surface permafrost on the Tibetan Plateau

To evaluate permafrost soil carbon age on the Tibetan Plateau, we collected surface permafrost below the active layer at 24 sites across a 1,000-km transect (see Qin et al.[2] for more detailed information). Briefly, at each site, five quadrats (1 m × 1 m) were set up at the corners and center of a 10 m × 10 m square plot. Within each quadrat, soil cores were drilled to a depth of 1.5–3.5 m according to the active layer thickness. Surface permafrost soils were obtained and transported back to the laboratory. Five soil replicates were mixed to form a composite sample, and its ^{14}C isotope was analyzed after air-drying and passing through a 2-mm sieve and removing roots and gravels. For more detailed information of ^{14}C isotopic analysis, please see Chen et al.[3]. Concisely, soil samples were first decalcified, freeze dried and combusted to obtain CO_2 gas. The evolved purified CO_2 was then frozen and reduced to graphite. After that, the radiocarbon was measured by an accelerator mass spectrometer (500kV tandem accelerator, NEC, USA) at Institute of Heavy Ion Physics, Peking University. Radiocarbon measurements were reported as conventional radiocarbon ages (years before present, yrs BP; where 0 yrs BP = AD 1950).

Supplementary Table 1. Physical and chemical properties of alpine thermokarst lakes on the Tibetan Plateau.

N	Permafrost regions	Grassland types	Lat (°N)	Long (°E)	Altitude (m)	Area (m^2)	Depth (m)	SAL (ppt)	ORP (mV)	pH	DO (mg L^{-1})	LSOC (mg g^{-1})	PCC (%)
1	MS	SM	34.5	99.2	4321	314.0±96.6	0.9±0.26	0.6±0.04	157.1±13.4	8.0±0.08	3.2±0.4	75.8	12.5±6.5
2	MS	AM	34.1	99.3	4405	889.6±371.5	1.2±0.19	0.2±0.01	239.3±13.6	8.0±0.08	4.2±0.2	111.1	0
3	MS	SM	34.8	99.0	4633	117.8±55.9	0.2±0.04	0.2±0.02	180.3±12.8	7.9±0.04	4.0±0.2	97.1	0
4	MS	AM	35.1	98.7	4419	565.6±216.4	0.7±0.22	0.4±0.07	144.4±4.4	8.2±0.03	3.6±0.2	87.5	0
5	MS	AS	34.8	98.4	4219	1247.2±527.9	0.3±0.02	0.8±0.06	164.2±11.2	9.3±0.22	5.3±0.2	7	0
6	MS	AS	34.6	98.0	4181	117.8±37.0	0.2±0.01	0.6±0.04	131.0±20.0	9.0±0.19	4.2±0.5	33.5	31.3±3.7
7	MS	AM	34.2	97.8	4576	1156.5±525.8	1.2±0.26	0.4±0.03	194.0±4.7	8.1±0.02	3.2±0.1	88.8	0
8	MS	AM	33.9	97.3	4508	295.4±113.3	0.4±0.04	1.0±0.03	206.3±14.2	7.9±0.06	3.0±0.2	23.4	0
9	MS	AM	34.2	96.0	4653	916.9±569.0	1.2±0.21	0.7±0.03	228.9±11.0	8.0±0.15	4.7±0.2	89.3	0
10	MS	AM	33.7	97.1	4437	846.0±262.1	1.6±0.72	0.3±0.01	219.3±14.8	7.7±0.09	3.3±0.4	155.4	5.0±4.3
11	BNZS	SM	34.9	94.7	4396	1497.0±705.9	1.5±0.12	0.3±0.01	197.7±19.4	8.2±0.09	4.0±0.3	53.3	32.5±5.5
12	BNZS	SM	32.8	95.0	4498	48.1±20.4	0.2±0.04	0.3±0.01	193.7±8.1	8.2±0.11	3.8±0.2	65	70.0±10.8
13	BNZS	AM	32.9	94.3	4768	1475.8±395.9	1.3±0.32	0.3±0.01	191.6±13.6	8.4±0.07	3.5±0.2	61.6	0
14	BNZS	AM	32.5	93.7	4746	143.3±56.9	0.3±0.03	0.4±0.01	204.6±4.1	8.7±0.09	4.3±0.3	101.1	22.5±7.4
15	BNZS	AM	31.9	93.1	4439	75.6±18.9	0.5±0.03	0.5±0.02	223.0±3.6	7.7±0.08	3.8±0.2	32.9	11.3±2.7
16	BNZS	SM	31.3	91.9	4497	128.5±54.6	0.4±0.04	0.6±0.03	217.5±8.4	8.5±0.16	4.2±0.1	20	13.8±3.3
17	BNZS	SM	31.5	92.2	4581	171.7±41.9	0.3±0.03	0.6±0.02	220.9±16.3	7.9±0.07	5±0.2	47.9	21.3±5.7
18	BNZS	SM	31.7	91.8	4620	158.0±33.5	0.4±0.07	0.5±0.01	257.6±3.0	8.4±0.10	4.5±0.1	80.1	8.8±2.7

N	Permafrost regions	Grassland types	Lat (°N)	Long (°E)	Altitude (m)	Area (m^2)	Depth (m)	SAL (ppt)	ORP (mV)	pH	DO (mg L^{-1})	LSOC (mg g^{-1})	PCC (%)
19	BNZS	AM	32.6	91.9	5014	328.6±180.8	0.4±0.06	0.4±0.01	166.8±6.2	8.0±0.02	3.5±0.2	11.8	22.5±13.3
20	BNZS	AS	33.1	91.9	4886	211.0±26.3	0.3±0.04	0.9±0.06	232.8±14.0	8.4±0.02	4.2±0.3	9.5	0
21	BNZS	AM	34.1	92.3	4725	151.1±37.6	0.4±0.04	0.5±0.05	195.6±22.7	8.5±0.16	5.5±0.3	9.7	50.0±20.3
22	BNZS	AM	34.8	92.9	4628	263.0±110.8	0.2±0.04	0.9±0.03	209.9±10.2	8.9±0.12	4.7±0.3	11.5	10.0±8.7
23	BNZS	SM	35.1	94.2	4406	338.5±93.6	0.2±0.02	0.9±0.01	202.6±2.4	8.8±0.24	6.5±0.4	12.9	57.5±10.4
24	BNZS	AS	32.2	91.7	4806	1597.1±961.9	0.4±0.09	0.6±0.02	245.6±15.7	8.4±0.13	4.6±0.3	14.1	12.5±10.8
25	BNZS	AS	35.1	93.0	4680	167.0±52.1	0.4±0.02	2.6±0.54	227.1±4.8	8.5±0.05	4.7±0.0	6	0
26	QS	SM	37.5	100.3	3820	38.3±4.9	0.3±0.01	0.3±0.01	206.3±19.8	7.4±0.04	3.0±0.2	165.9	3.3±1.2
27	QS	SM	38.7	99.3	3443	1287.9±754.3	0.2±0.02	0.4±0.03	183.9±7.9	8.2±0.06	6.8±0.7	22.7	33.8±4.5
28	QS	SM	37.8	101.1	3600	1012.0±526.6	1.7±0.17	0.3±0.01	155.6±23.9	7.8±0.17	3.8±0.4	101.2	0
29	QS	AM	38.0	100.8	3279	3112.8±1909.2	0.3±0.01	1.9±0.19	218.1±6.9	9.3±0.06	6.5±0.4	60.3	34.5±7.5
30	QS	SM	37.7	100.8	3618	699.4±576.3	0.9±0.4	0.3±0.02	131.5±13.1	7.8±0.07	3.0±0.3	160	8.8±7.6

N represents lake number; MS, Madoi section; BNZS, Budongquan-Nagqu-Zadoi section; QS, Qilian section. AS, alpine steppe; AM, alpine meadow; SM, swamp meadow; Lat, latitude; Long, longitude; SAL, salinity; ORP, oxidation-reduction potentiality; DO, dissolved oxygen concentration; LSOC, lake sediment organic carbon; PCC, plant community coverage. The righthand sides of the plus or minus signs represent standard error (SE) of four sampling lakes at each cluster. SE is not shown for LSOC since it is only measured in one thermokarst lake at each cluster.

Supplementary Table 2. Stable and radiocarbon isotopic composition of CH_4 and CO_2 in the bubble samples from alpine thermokarst lakes on the Tibetan Plateau.

N	$\delta^{13}C$-CH_4 (‰)	α_C	$\Delta^{14}C$-CH_4 (‰)	^{14}C-CH_4 Age (yrs BP)	$\delta^{13}C$-CO_2 (‰)
1	-63.8	1.058	-26.7	215	-9.8
2	-71.5	1.076	18.8	-145	-0.49
3	-76.7	1.068	3.6	-25	-14.1
4	-74.2	1.070	46.4	-360	-9.2
5	-67.8	1.064	nd	nd	-8.5
6	-62.2	1.054	3.8	-25	-11.6
7	-72.4	1.068	-56.7	470	-8.9
8	-83.4	1.068	-11.9	95	-21.0
9	-77.2	1.074	-30.2	245	-8.6
10	-77.6	1.079	-8.2	65	-4.4
11	-73.3	1.063	20.6	-160	-15.4
12	-71.5	1.055	-40.8	335	-20.4
13	-64.6	1.065	21	-160	-4.2
14	-66.9	1.050	10.7	-80	-20.1
15	-81.2	1.065	27.4	-210	-21.8
16	-72.4	1.053	nd	nd	-22.9
17	-83.4	1.068	23.9	-185	-21.4
18	-68.9	1.056	nd	nd	-17.1
19	-79.0	1.071	-76.9	645	-13.4
20	-78.0	1.064	-7	55	-19.1
21	-69.6	1.056	nd	nd	-17.1
22	-66.1	1.052	4.6	-30	-17.7
23	-66.6	1.060	-377.1	3805	-10.4
24	-83.1	1.071	nd	nd	-17.6
25	nd	nd	nd	nd	nd
26	-71.7	1.056	16.2	-125	-19.7
27	-69.4	1.064	-14.8	120	-9.4
28	-75.8	1.071	-112.3	955	-10.6
29	-67.9	1.064	-208.2	1875	-8.1
30	-67.5	1.067	-54.1	445	-5.4

N represents lake number. The α_C values indicate the pathway of CH_4 production, with $\alpha_C > 1.055$ suggesting that CH_4 is mainly produced by CO_2 reduction, and $\alpha_C < 1.055$ suggesting that CH_4 is produced increasingly by acetate fermentation. nd indicates that the corresponding values are not determined due to the limited gas samples.

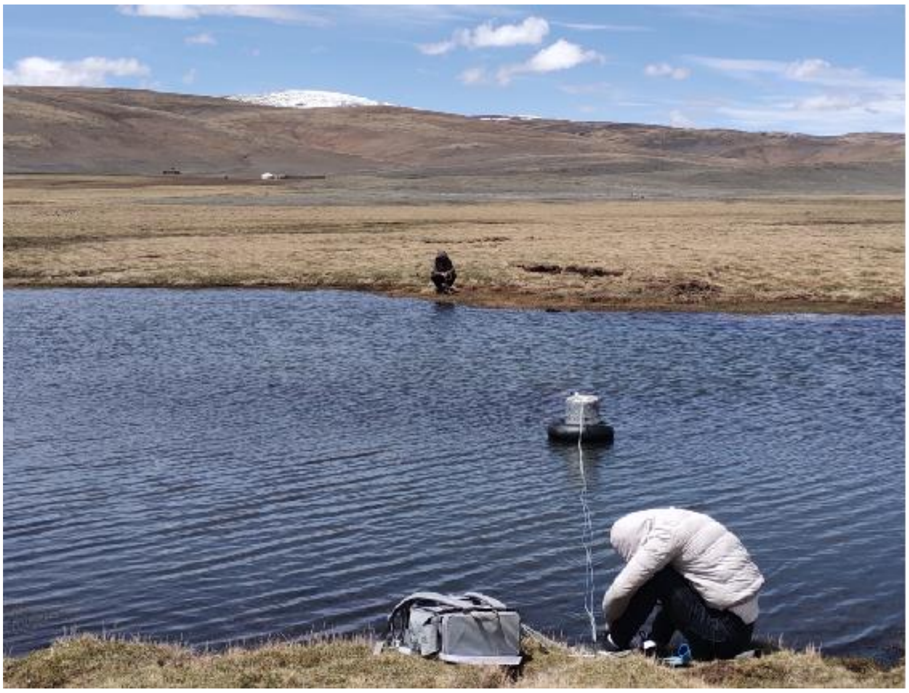

Supplementary Figure 1. Photograph of *in-situ* flux measurement using a near-infrared laser CO_2/CH_4 analyzer. Photograph is taken by Guibiao Yang.

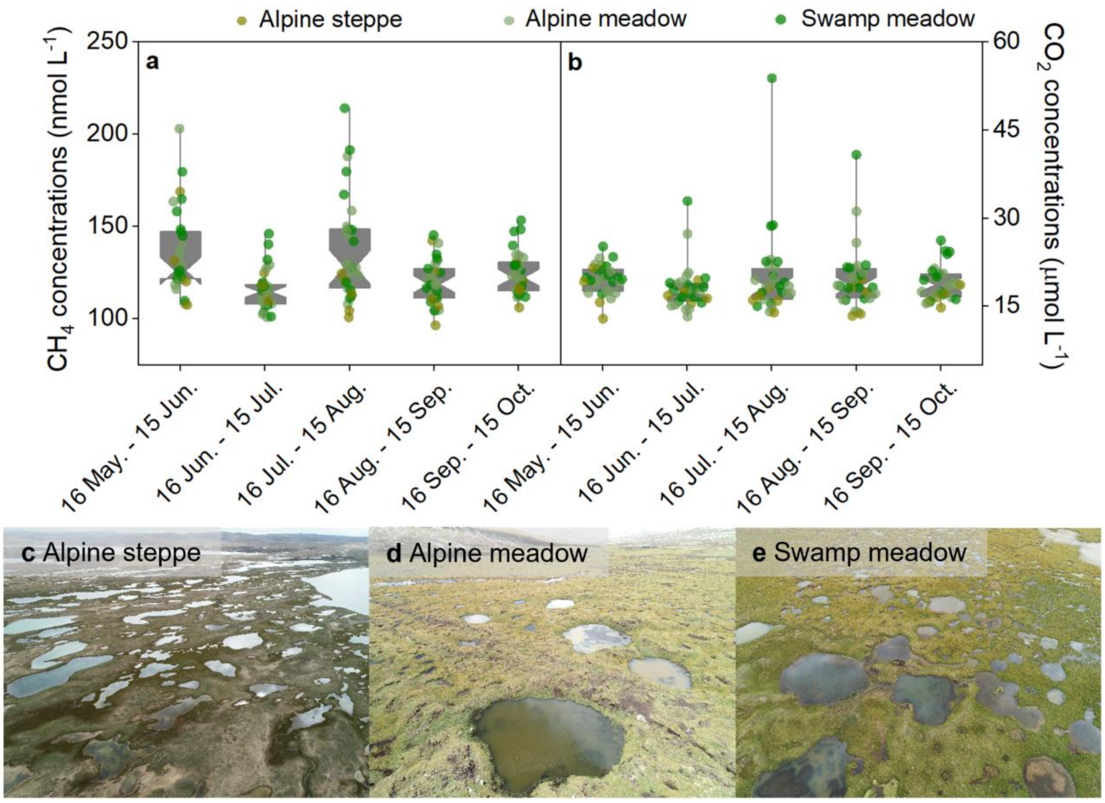

Supplementary Figure 2. Seasonal dynamics of CH_4 (a) and CO_2 (b) concentrations from alpine thermokarst lakes on the Tibetan Plateau, and landscapes of thermokarst lakes distributed in various grassland types (c-e). Box plots present the 25th and the 75th quartile (interquartile range), and whiskers indicate the data range among 30 clusters of thermokarst lakes sampled in this study ($n = 30$). The notches are the medians with 95% confidence intervals. Observed values are shown as dots. The different colors represent thermokarst lakes distributed in various grassland types (alpine steppe, alpine meadow and swamp meadow). Photographs in panels (c-e) are taken by Guibiao Yang.

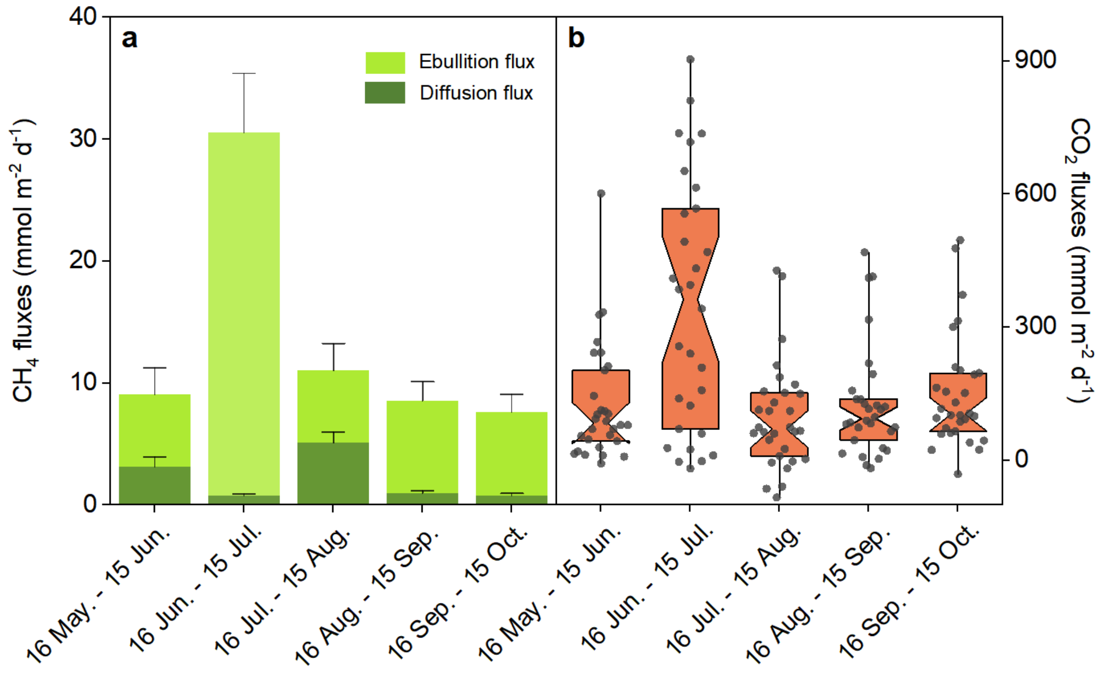

Supplementary Figure 3. Seasonal dynamics of CH_4 (a) and CO_2 (b) fluxes from alpine thermokarst lakes on the Tibetan Plateau. In panel (a), CH_4 fluxes are color coded according to different transportation pathways (ebullition and diffusion). Data show the average CH_4 and CO_2 fluxes across 30 clusters of thermokarst lakes in different sampling periods. Data are presented as mean values ± standard error (n = 30). In panel (b), box plots present the 25th and the 75th quartile (interquartile range), and whiskers indicate the data range among 30 clusters of thermokarst lakes sampled in this study. The notches are the medians with 95% confidence intervals. Observed values are shown as black dots (n = 30).

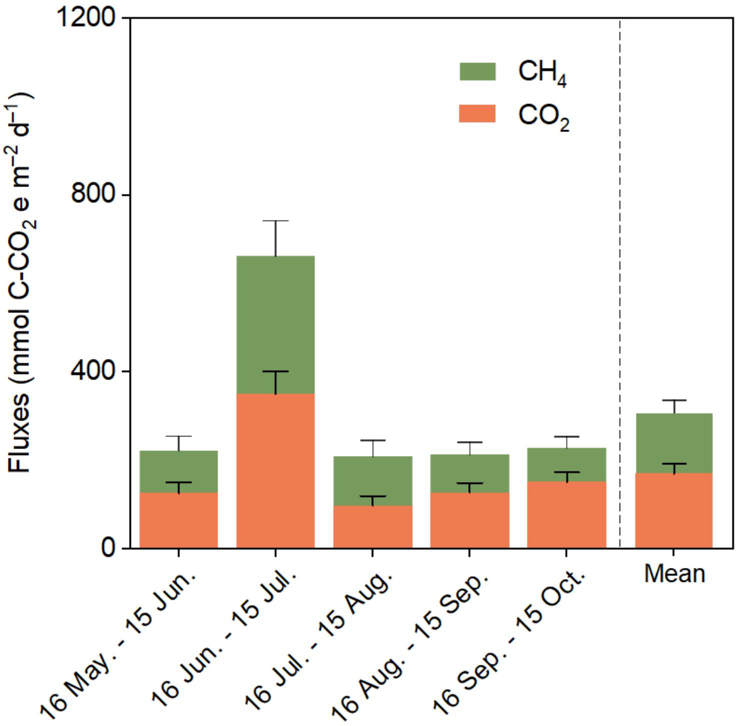

Supplementary Figure 4. Seasonal dynamics of carbon dioxide equivalent (CO_2-e) fluxes from alpine thermokarst lakes on the Tibetan Plateau. The lefthand side of the dotted line shows the seasonal dynamics of gas fluxes, while the righthand side illustrates the average gas fluxes during the growing season. CO_2-e fluxes are color coded for CH_4 and CO_2. Data are presented as mean values ± standard error (n = 30).

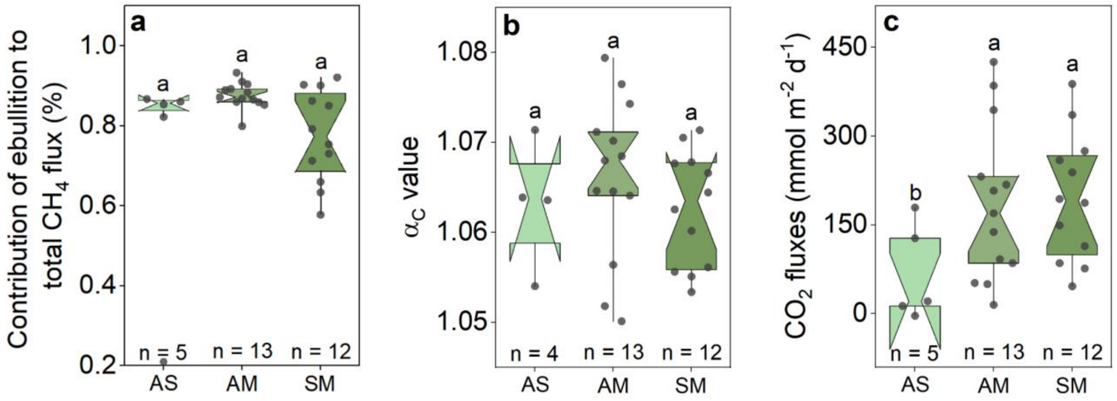

Supplementary Figure 5. Comparisons of contribution of ebullition to total CH_4 fluxes (a, $p = 0.072$), α_C value (b, $p = 0.516$) and CO_2 fluxes (c, $p = 0.002$) from thermokarst lakes located in various grassland types on the Tibetan Plateau. AS, AM and SM represent alpine steppe, alpine meadow and swamp meadow, respectively. Box plots present the 25th and the 75th quartile (interquartile range), and whiskers indicate the data range among thermokarst lakes located in AS, AM and SM, respectively. The notches are the medians with 95% confidence intervals. Observed values are shown as black dots, and numbers shown in each panel represent sample sizes. Different letters represent no significant differences among thermokarst lakes located in the various grassland types (One-way ANOVAs with two-sided Tukey's HSD multiple comparisons, $p < 0.05$).

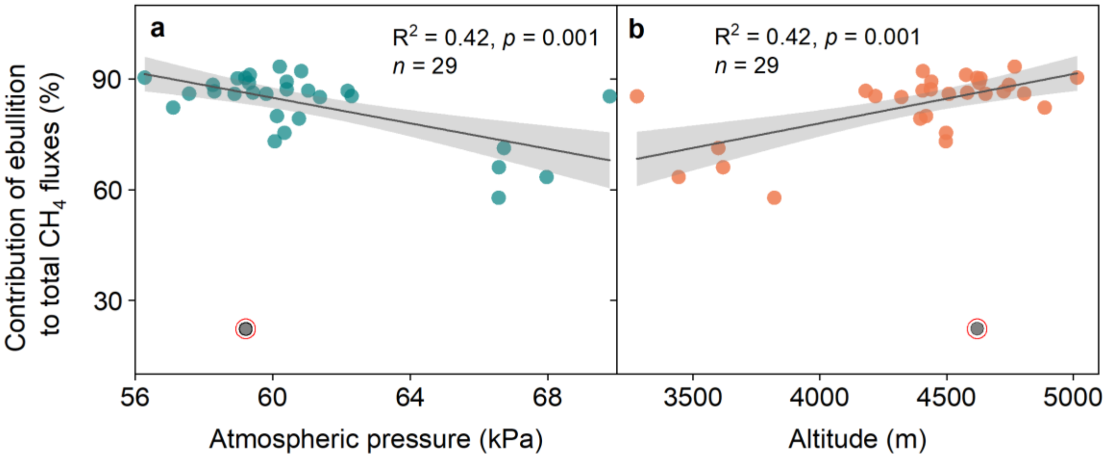

Supplementary Figure 6. Relationships of the contribution of ebullition to total CH_4 fluxes with atmospheric pressure (a) and altitude (b). Outliers (grey points) were excluded from the linear regression analysis based on Boxplot Procedures. Gray bands depict the 95% confidence intervals around the regression lines (centers of error bands).

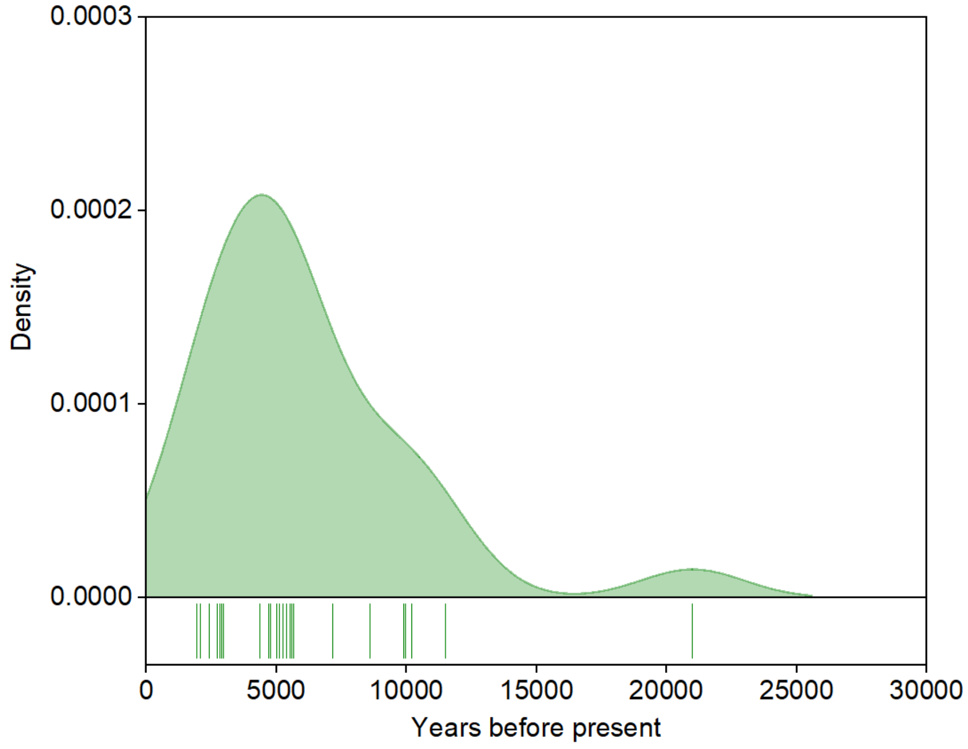

Supplementary Figure 7. Density distribution of radiocarbon age from surface permafrost on the Tibetan Plateau. The data were derived from a large-scale field investigation along a 1,000-km permafrost transect on the Tibetan Plateau (Supplementary Note 2). The line indicates surface permafrost radiocarbon age at each sampling site ($n = 24$).

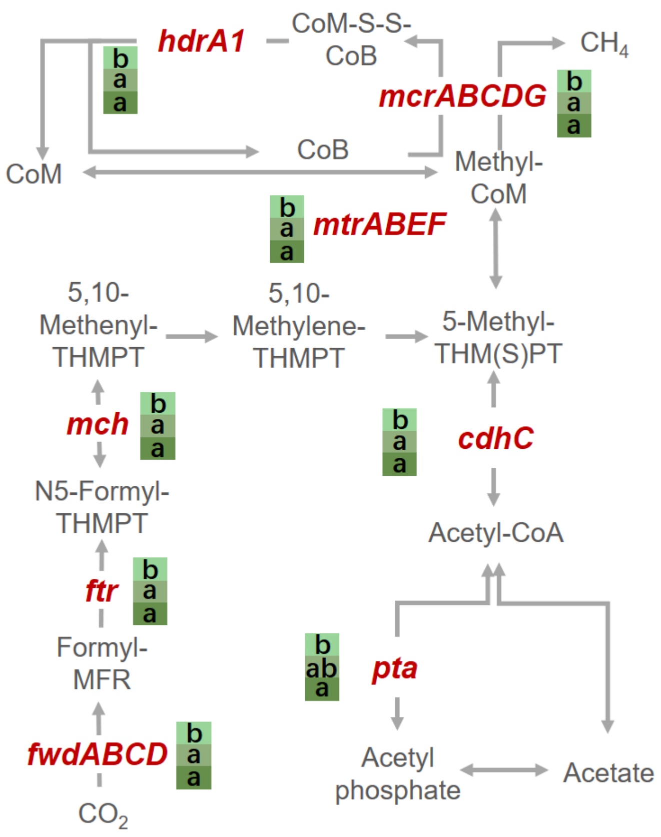

Supplementary Figure 8. Differences of functional genes involved in methanogenesis among thermokarst lakes located in alpine steppe (light green), alpine meadow (medium green), and swamp meadow (dark green). Metabolic pathways were chosen based on KEGG modules M00567 and M00357. Red letters represent the genes which were significantly different among thermokarst lakes distributed in the three ecosystem types. *hcr*, Heterodisulfide reductase subunit A; *mcr*, Methyl-coenzyme reductase; *mtr*, Tetrahydromethanopterin S-methyltransferase; *mch*, Methenyltetrahydromethanopterin cyclohydrolase; *ftr*, Formylmethanofuran-tetrahydromethanopterin N-formyltransferase; *fwd*, Formylmethanofuran dehydrogenase; *cdh*; Acetyl-CoA decarbonylase/synthase complex; *pta*, Phosphate acetyltransferase. Significant differences are denoted by different letters (One-way ANOVAs with two-sided Tukey's HSD multiple comparisons, $p < 0.05$).

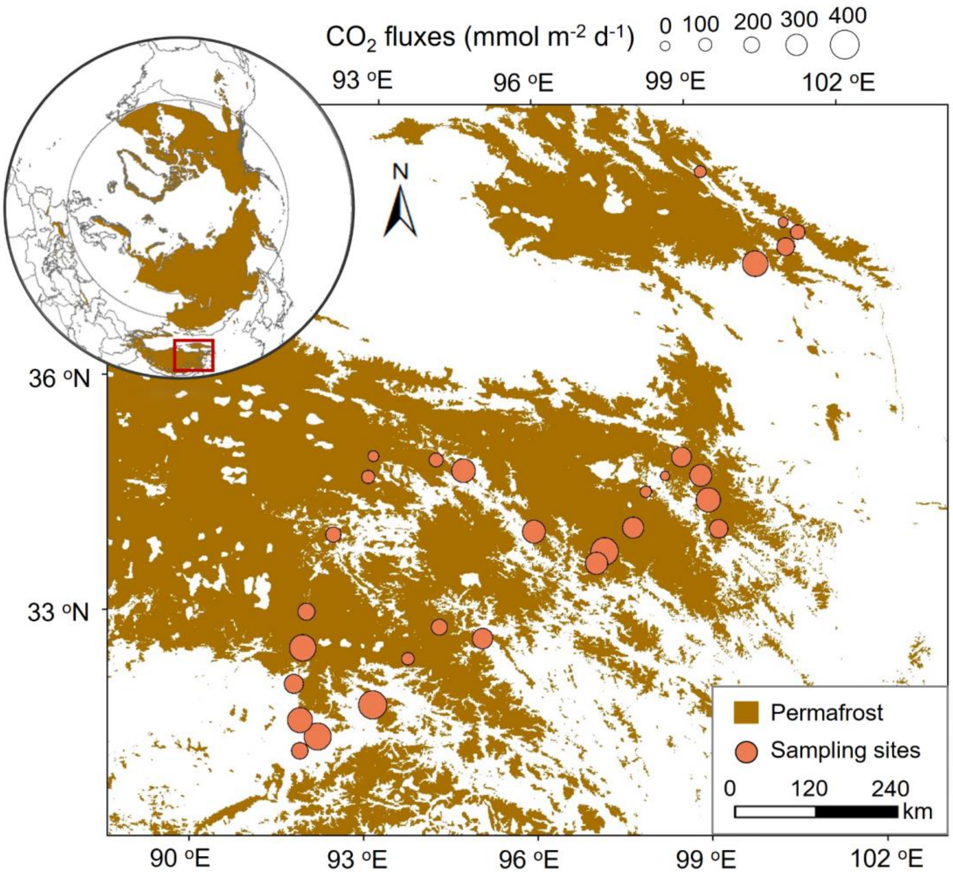

Supplementary Figure 9. Spatial distribution of CO_2 fluxes across alpine thermokarst lakes on the Tibetan Plateau. Bubble size is proportional to the value of CO_2 fluxes, with larger size representing higher values. The background permafrost maps of the Northern Hemisphere and the Tibetan Plateau are derived from the National Snow & Ice Data Center[4] (https://nsidc.org/data/ggd318/versions/2) and Zou *et al.*[5] (https://tc.copernicus.org/articles/ 11/2527/2017/) / CC BY (https://creativecommons.org/licenses/by/3.0/), respectively.

Supplementary References

1. Holgerson, M. A. & Raymond, P. A. Large contribution to inland water CO_2 and CH_4 emissions from very small ponds. *Nat Geosci.* **9**, 222-226 (2016).
2. Qin, S. et al. Temperature sensitivity of permafrost carbon release mediated by mineral and microbial properties. *Sci. Adv*. **7**, eabe3596 (2021).
3. Chen, L. et al. Soil carbon persistence governed by plant input and mineral protection at regional and global scales. *Ecol Lett*. **24**, 1018-1028 (2021).
4. Brown, J. et al. *Circum-arctic map of permafrost and ground ice conditions, Version 2*. (National Snow & Ice Data Center, 2002).
5. Zou, D. et al. A new map of permafrost distribution on the Tibetan Plateau. *Cryosphere,* **11,** 2527-2542 (2017).
