# Supplementary material for: Characteristics of methane emissions from alpine thermokarst lakes on the Tibetan Plateau
Source: Nat Commun. 2023 May 30;14:3121. doi: 10.1038/s41467-023-38907-6 (PMC10229571; doi:10.1038/s41467-023-38907-6)
Supplement: Supplementary file 3 — Description of Additional Supplementary Files [file 41467_2023_38907_MOESM3_ESM.pdf]

Description of Additional Supplementary Files:

Supplementary Movie 1: Video of visible micro-bubbles rising from sediment to the water surface. This video was shot by Guibiao Yang at the shore of a thermokarst lake located in Qumarlêb County, Qinghai Province, China.
