# Supplementary material for: Characteristics of methane emissions from alpine thermokarst lakes on the Tibetan Plateau
Source: Nat Commun. 2023 May 30;14:3121. doi: 10.1038/s41467-023-38907-6 (PMC10229571; doi:10.1038/s41467-023-38907-6)
Supplement: Supplementary file 5 — Reporting Summary [file 41467_2023_38907_MOESM5_ESM.pdf]

nature portfolio

Corresponding author(s): Yuanhe Yang

Last updated by author(s): May 1, 2023

Reporting Summary

Nature Portfolio wishes to improve the reproducibility of the work that we publish. This form provides structure for consistency and transparency in reporting. For further information on Nature Portfolio policies, see our Editorial Policies and the Editorial Policy Checklist.

Statistics

For all statistical analyses, confirm that the following items are present in the figure legend, table legend, main text, or Methods section.

n/a	Confirmed	
☐	☒	The exact sample size (*n*) for each experimental group/condition, given as a discrete number and unit of measurement
☐	☒	A statement on whether measurements were taken from distinct samples or whether the same sample was measured repeatedly
☐	☒	The statistical test(s) used AND whether they are one- or two-sided<br>*Only common tests should be described solely by name; describe more complex techniques in the Methods section.*
☒	☐	A description of all covariates tested
☐	☒	A description of any assumptions or corrections, such as tests of normality and adjustment for multiple comparisons
☐	☒	A full description of the statistical parameters including central tendency (e.g. means) or other basic estimates (e.g. regression coefficient) AND variation (e.g. standard deviation) or associated estimates of uncertainty (e.g. confidence intervals)
☐	☒	For null hypothesis testing, the test statistic (e.g. *F*, *t*, *r*) with confidence intervals, effect sizes, degrees of freedom and *P* value noted<br>*Give P values as exact values whenever suitable.*
☒	☐	For Bayesian analysis, information on the choice of priors and Markov chain Monte Carlo settings
☒	☐	For hierarchical and complex designs, identification of the appropriate level for tests and full reporting of outcomes
☐	☒	Estimates of effect sizes (e.g. Cohen's *d*, Pearson's *r*), indicating how they were calculated

Our web collection on statistics for biologists contains articles on many of the points above.

Software and code

Policy information about availability of computer code

Data collection	No software was used in this study for data collection.
Data analysis	Permafrost maps were generated using ArcGIS v.10.3. Metagenomics data were analyzed by software packages included SOAPnuke v1.5.2, SOAP v2.22, Megahit v.1.2.9, Prodigal v.2.6.3, CD-HIT-EST v.4.8.1, DIAMOND v.2.0.9, Salmon v.1.5.1, Kraken2 v.2.1.2, Prodigal v.2.6.3, HMMER v.3.3.2, MUSCLE v.3.8.31, FastTree v.2.1.11, Salmon v.1.5.1. against EggNOG v.5.0, KEGG, mini and Pfam reference databases. The species composition was visualized by the GraPhlAn v.0.9.7. All statistical analyses were performed using the software package R v.3.6.2. Details were reported in the Methods section.

For manuscripts utilizing custom algorithms or software that are central to the research but not yet described in published literature, software must be made available to editors and reviewers. We strongly encourage code deposition in a community repository (e.g. GitHub). See the Nature Portfolio guidelines for submitting code & software for further information.

Data

Policy information about availability of data

All manuscripts must include a data availability statement. This statement should provide the following information, where applicable:

- Accession codes, unique identifiers, or web links for publicly available datasets
- A description of any restrictions on data availability
- For clinical datasets or third party data, please ensure that the statement adheres to our policy

All data supporting the findings are available in the Figshare data repository (https://doi.org/10.6084/m9.figshare.22743968) and Supplementary Information. The

nucleotide sequences generated by metagenome sequencing have been deposited in the NCBI database (ncbi.nlm.nih.gov/bioproject/?term=PRJNA942440).

Human research participants

Policy information about studies involving human research participants and Sex and Gender in Research.

Reporting on sex and gender	This research is not related to sex-and gender-. The information about sex- and gender- has not been collected in this study.
Population characteristics	This study does not involve population.
Recruitment	This study does not involve population.
Ethics oversight	This study does not involve human research, and there is no human research related organization involved.

Note that full information on the approval of the study protocol must also be provided in the manuscript.

Field-specific reporting

Please select the one below that is the best fit for your research. If you are not sure, read the appropriate sections before making your selection.

☐ Life sciences ☐ Behavioural & social sciences ☒ Ecological, evolutionary & environmental sciences

For a reference copy of the document with all sections, see nature.com/documents/nr-reporting-summary-flat.pdf

Ecological, evolutionary & environmental sciences study design

All studies must disclose on these points even when the disclosure is negative.

Study description	This study provided a comprehensive understanding on the spatial patterns, sources and microbial characteristics of CH4 emissions from 120 thermokarst lakes in 30 clusters along a 1,100 km transect on the Tibetan Plateau.
Research sample	30 representative sampling clusters of thermokarst lakes were selected along a 1,100 km transect on the Tibetan Plateau. The 30 clusters of thermokarst lakes were evenly located in the representative permafrost regions, and located in the alpine grasslands as they represent ~80% thermokarst lakes. Gas, water and sediment samples were obtained from thermokarst lakes at each cluster.
Sampling strategy	Sampling strategy was adopted to maximize the times and number of flux measurements of thermokarst lakes so as to well characterize greenhouse gas emissions. Specifically, at each cluster, four lakes were selected to consider the spatial variability of carbon fluxes among thermokarst lakes. At each lake, 4 to 6 sampling locations was determined by area of lakes, instrumentation performance and field activities which limited the number of samples collected in our study. Each lake was sampled five times at monthly intervals during the ice-free period due to the time limitations. Carbon flux during the ice-free period were only measured due to the harsh field conditions during the ice period.
Data collection	In-situ total carbon fluxes were determined using an opaque lightweight floating chamber equipped with a closed loop to a near-infrared laser CH4/CO2 analyzer (GLA231-GGA, ABB., Canada). Specifically, the floating chamber was flushed with ambient air for ~10 sec before each measurement. CH4 and CO2 concentrations in the chamber were continuously recorded at an interval of 1 sec after an equilibration period. CH4 and CO2 fluxes were determined as the slope of the linear relationship between their concentrations and measurement time. In addition, CH4 and CO2 concentrations were recorded with the CH4/CO2 analyzer (GLA231-GGA, ABB., Canada). Wind speed and atmospheric temperature with a portable anemometer (Testo 480, Testo SE & Co. KGaA, Lenzkirch, Germany). Air pressure, water temperature, oxidation-reduction potentiality, pH and dissolved oxygen concentration were measured with a portable multiparameter water quality instrument (ProSolo Digital Water Quality Meter, Yellow Springs Instrument, Brannum Lane, USA). Paper, pen, and computers were used for these recoding. The detailed data collection procedure was described in the Methods section. Guibiao Yang, Zhihu Zheng, Luyao Kang and Shuqi Qin were present during data collection.
Timing and spatial scale	Carbon fluxes were measured once a month during the open water season (from mid-May to mid-October, 2021) to explore seasonal variation. Due to the high cost of laboratory analyses (such as radio-carbon isotopic analyses and metagenomic sequencing), gas and sediment samples were collected once during mid-July to mid- August, 2021. Sampling was performed across a regional scale (Latitude: 31-39oN, longitude: 91-101oE). Detailed location is available in the supplementary Table 1.
Data exclusions	No data were excluded from the analyses.
Reproducibility	Replicate measurements were taken for carbon fluxes (five times at monthly intervals during the ice-free period). Reproducibility of experimental design is not relevant, per se, because the ambient conditions are always changing and measurements will be different despite of the same sampling lake.
Randomization	We collected 120 thermokarst lakes at 30 clusters evenly located in three representative permafrost regions on the Tibetan Plateau. Each thermokarst lake was sampled five times during the ice-free period from mid-May to mid-October. At each cluster, the sampling was taken at multiple locations within multiple lakes, and the gas and sediment samplings were randomly collected and well-mixed in each lake. This strategy increases the area of the sampling and the randomization factors of any sample, which improves the reliability of the generated dataset.

Blinding	Not relevant because this was an observational study.

Did the study involve field work? ☒ Yes ☐ No

Field work, collection and transport

Field conditions	Field conditions were harsh in this study region, such as low temperature, oxygen limitation, and traffic inconvenience. Please see "Study area" and "Flux and environmental measurements" in the Method section, and Figure 1d-h and Supplementary Table 1 for details.
Location	Latitude: 31-39oN, longitude: 91-101oE. Elevation: 3279-5014m. Water depth: 0.2-1.7m. The 30 clusters are located in three representative permafrost regions (10 sites in the Madoi section on the eastern plateau, 15 sites in the Budongquan-Nagqu-Zadoi section, and 5 sites in the Qilian section on the northeastern plateau in the central part of the plateau). Please see Supplementary Table 1 for details.
Access & import/export	The access to Three-River-Source National Park was granted by Three-River-Source National Park Management Bureau (TNPMB) during the sampling period from May to November. Sampling collection and transport were conducted under the authorization of the TNPMB.
Disturbance	The floating chambers used in this study were covered with aluminum foil to reflect the sunlight and minimize internal heating. The chamber was then pulled into its sampling location with a rope to avoid the need to enter the lake and potentially disturb sediment and gas release. 20s were equilibrated before recording concentrations of CH_4 or CO_2 to eliminate the disturbance of surface boundary layer induced by chamber deployment. These efforts were made to minimize the disturbance when measuring fluxes.

Reporting for specific materials, systems and methods

We require information from authors about some types of materials, experimental systems and methods used in many studies. Here, indicate whether each material, system or method listed is relevant to your study. If you are not sure if a list item applies to your research, read the appropriate section before selecting a response.

Materials & experimental systems

n/a	Involved in the study	
☒	☐	Antibodies
☒	☐	Eukaryotic cell lines
☒	☐	Palaeontology and archaeology
☒	☐	Animals and other organisms
☒	☐	Clinical data
☒	☐	Dual use research of concern

Methods

n/a	Involved in the study	
☒	☐	ChIP-seq
☒	☐	Flow cytometry
☒	☐	MRI-based neuroimaging
